# Spinal Distortions

**Published:** 1842-07-30

**Authors:** 


*Spinal Distortions. Division of the Muscles of the Back.*—In an article in the "Gazette Médicale," M. Guerin endeavours to refute the objections raised by M. Bouvier against his theory of the dependancy of spinal distortions on muscular contraction, and the applicability of tenotomy to their cure. For the present we will simply notice M. Guerin's theory, reserving to some future number a summary of the different theories on this class of affections. According to M. Guerin spinal distortions should be classed with club-foot, wry-neck, &c., and that as the muscles of the foot, leg, knee, &c., by their contractions produce certain deformities, which, arising from the same cause, perverted muscular action, present the same general character, and require for their relief the same operation, division of the contracted muscles; so also curvature of the spine may be considered as the club-foot of the back, depending on the contracted state of the muscles of this region, and requiring for its cure their division.—*London Lancet.* May 14, 1842.

